# Pretreatment Body Composition Parameters and HALP Score in Relation to Pathological Complete Response in HER2-Positive Breast Cancer Receiving Neoadjuvant Systemic Therapy

**DOI:** 10.3390/jcm15207927

**Published:** 2026-10-14

**Authors:** Neşe Alyıldız, Filiz Taşcı, Eda Beykoz Çetin, Esra Aydın, Eyyüp Ayas, Oğuzhan Kesen

**Affiliations:** 1 Department of Medical Oncology, Faculty of Medicine, Recep Tayyip Erdoğan University Training and Research Hospital, 53020 Rize, Türkiye; 2 Department of Radiology, Faculty of Medicine, Recep Tayyip Erdoğan University Training and Research Hospital, 53020 Rize, Türkiye

**Keywords:** HER2-positive breast cancer, pathological complete response, body composition, skeletal muscle index, myosteatosis, HALP score

## Abstract

**Background/Objectives:** Pathological complete response (pCR) is an important early endpoint in HER2-positive breast cancer treated with neoadjuvant therapy. Although tumor biology is a major determinant of response, the potential contribution of host-related factors such as body composition and systemic inflammatory-nutritional status remains uncertain. This study evaluated the associations of pretreatment skeletal muscle index (SMI), myosteatosis, an imaging-defined low-SMI obesity phenotype, and hemoglobin-albumin-lymphocyte-platelet (HALP) score with pCR. **Methods:** This retrospective single-center study included 114 patients with HER2-positive disease documented on pretreatment biopsy who received neoadjuvant systemic therapy followed by definitive surgery. CT-based body-composition measurements at the L3 level were available in 88 patients. SMI, skeletal muscle radiodensity, myosteatosis, an imaging-defined low-SMI obesity phenotype, and pretreatment HALP score were assessed. Factors associated with pCR were evaluated using Firth penalized logistic regression. Two multivariable models incorporated body-composition variables, HALP, hormone receptor status, simplified clinical T and N categories, HER2-treatment group, age, and Ki-67. Final multivariable analyses included 80 complete cases. **Results:** pCR was achieved in 59 of 114 patients (51.8%). Hormone receptor-negative tumors had a higher pCR rate than hormone receptor-positive tumors (74.4% vs. 40.0%; *p* < 0.001). Pretreatment SMI, myosteatosis, the imaging-defined low-SMI obesity phenotype, and HALP were not significantly associated with pCR in univariable analyses. In multivariable analyses, SMI and myosteatosis were not independently associated with pCR, whereas hormone receptor positivity remained associated with lower pCR odds. The treatment-group coefficient indicated lower adjusted pCR odds with trastuzumab alone than with dual HER2 blockade; however, this estimate was based on sparse complete-case subgroup data and should not be interpreted as a comparative treatment-effect estimate. Muscle attenuation and myosteatosis classification differed significantly by CT acquisition type, whereas SMI did not. **Conclusions:** Pretreatment SMI, myosteatosis, the imaging-defined low-SMI obesity phenotype, and HALP were not significantly associated with pCR in this cohort. Hormone receptor status remained strongly associated with response. Treatment-related estimates and attenuation-based body-composition measures should be interpreted cautiously because of sparse subgroup data, treatment-era confounding, and imaging heterogeneity. Larger prospective studies with standardized imaging are warranted.

## 1. Introduction

HER2-positive breast cancer is defined by overexpression and/or amplification of human epidermal growth factor receptor 2 (HER2) and represents a biologically aggressive breast cancer subtype. In early and locally advanced disease, neoadjuvant HER2-directed therapy has become a central component of treatment, particularly with the incorporation of trastuzumab and pertuzumab [1,2,3]. Pathological complete response (pCR), defined in this study as ypT0/is ypN0, is an established early measure of treatment efficacy and is associated with improved long-term outcomes, especially in more aggressive biological subtypes [4,5,6]. Nevertheless, a substantial proportion of patients have residual invasive disease after neoadjuvant therapy, underscoring the need for additional markers that may refine assessment of treatment response.

Host-related characteristics may contribute to this heterogeneity in response. Nutritional reserve, systemic inflammatory activity, and body composition have therefore attracted increasing interest in breast cancer research [7,8]. Routine staging CT provides a practical opportunity to quantify skeletal muscle without additional imaging. Skeletal muscle index (SMI), calculated by normalizing L3 skeletal muscle area to height squared, is widely used as a marker of muscle quantity. Low muscle mass has been associated with treatment toxicity and adverse outcomes in breast cancer, and its possible relationship with neoadjuvant response has recently been explored [7,8,9,10]. Some studies in HER2-positive and triple-negative breast cancer have suggested lower pCR rates among patients with reduced pretreatment SMI, although the evidence remains inconsistent [9,10].

Muscle quantity alone does not fully describe skeletal muscle phenotype. Skeletal muscle radiodensity (SMD), measured in Hounsfield units (HU), provides an imaging-based estimate of muscle quality; lower attenuation values generally indicate greater intramuscular lipid infiltration and are commonly used to define myosteatosis [11,12,13]. CT-derived muscle and adiposity measures have shown prognostic relevance in nonmetastatic breast cancer [11], and muscle radiodensity has also been evaluated in metastatic disease [12]. Myosteatosis may therefore capture metabolic and structural changes that are not reflected by muscle area alone [13]. However, its association with pCR after neoadjuvant HER2-targeted therapy remains insufficiently characterized.

An imaging-defined low-SMI obesity phenotype may represent another body-composition pattern of potential interest by combining reduced skeletal muscle quantity with obesity. Because BMI cannot distinguish lean tissue from fat mass, it may fail to identify clinically relevant differences in body composition [11,14]. Contemporary definitions of sarcopenic obesity incorporate muscle function in addition to body-composition criteria [15]; therefore, in the absence of muscle-strength or physical-performance measures, the present study uses the more conservative term imaging-defined low-SMI obesity phenotype.

Blood-based composite indices provide an additional approach to characterizing systemic inflammatory and nutritional status. The hemoglobin-albumin-lymphocyte-platelet (HALP) score combines hemoglobin and albumin concentrations with circulating lymphocyte and platelet counts [16]. Its prognostic value has been investigated in breast cancer [17,18,19], while HALP-derived indices have more recently been studied in the neoadjuvant HER2-positive setting [20]. These observations support further evaluation of whether pretreatment inflammatory-nutritional status is related to treatment response.

Few studies have assessed skeletal muscle quantity, muscle quality, obesity-related body composition, and inflammatory-nutritional status concurrently in patients with HER2-positive breast cancer receiving neoadjuvant therapy. Accordingly, we evaluated the associations of pretreatment SMI, myosteatosis, the imaging-defined low-SMI obesity phenotype, and HALP with pCR. The study was designed as an exploratory observational association analysis rather than as a clinically deployable treatment-selection prediction model.

## 2. Materials and Methods

### 2.1. Study Design and Patient Population

This retrospective single-center cohort study was conducted at Recep Tayyip Erdoğan University Training and Research Hospital. Patients treated between March 2018 and December 2025 were screened. Eligible patients were aged ≥18 years, had nonmetastatic HER2-positive breast cancer documented on pretreatment biopsy, received neoadjuvant systemic therapy followed by definitive breast surgery, and had postoperative pathological findings available for assessment of pathological complete response (pCR). HER2 status was assigned according to the ASCO/CAP criteria in effect at the time of pathological assessment.

This retrospective observational cohort study was reported in accordance with the Strengthening the Reporting of Observational Studies in Epidemiology (STROBE) statement [21].

The December 2025 cutoff represented the upper limit of the retrospective clinical treatment period and was selected to allow sufficient time for completion of neoadjuvant therapy, definitive surgery, and availability of postoperative pathology. Patients with distant metastatic disease at presentation, a synchronous second primary malignancy, or insufficient clinical or pathological information to determine the primary endpoint were excluded.

A total of 120 patients were initially identified. Six patients who were HER2-negative on pretreatment biopsy and were found to be HER2-positive only in residual tumor at surgery were excluded from the primary cohort because inclusion of these patients would introduce outcome-dependent selection. The revised primary cohort therefore comprised 114 patients with pretreatment HER2-positive disease.

Clinical, pathological, treatment, laboratory, and imaging information was obtained retrospectively from institutional records. Availability of an adequate pretreatment CT image at the L3 level was required only for body-composition analyses. Of the 114 patients in the primary clinical cohort, 88 had evaluable pretreatment CT or PET/CT images for body-composition assessment. Patients without technically suitable L3 imaging remained eligible for analyses not requiring body-composition variables. Final multivariable analyses were performed using 80 complete cases with available data for all included covariates. The study selection process and analysis cohorts are summarized in Figure 1.

### 2.2. Data Collection and Clinicopathological Variables

Demographic, tumor-related, and treatment variables were abstracted from electronic medical records, pathology reports, and oncology treatment documentation. Variables included age, menopausal status, BMI, clinical T and N categories, overall stage, histology, grade, hormone receptor status, Ki-67, HER2 findings, neoadjuvant regimen, HER2-targeted treatment exposure, and postoperative pathological response.

Pretreatment BMI was calculated from recorded weight and height as kg/m^2^. For categorical analyses, BMI was primarily evaluated as <30 versus ≥30 kg/m^2^.

Clinical T and N categories were based on pretreatment physical examination and imaging. Overall clinical stage was assigned according to the AJCC staging system applicable during the treatment period.

ER and PR status was obtained from pretreatment biopsy pathology. Hormone receptor positivity was defined as positivity for either ER or PR; tumors negative for both markers were classified as hormone receptor-negative. HER2 assessment relied on immunohistochemistry and, when required, in situ hybridization according to contemporaneous ASCO/CAP recommendations.

Pretreatment Ki-67 was recorded as the percentage of positively stained tumor nuclei and analyzed as <30% versus ≥30% in the final regression models. Histological grade was recorded when available, but because grade was missing in nearly half of the cohort, it was not included in the principal multivariable models.

The chemotherapy backbone and HER2-directed treatment exposure were analyzed as separate variables. The principal HER2-treatment comparison focused on trastuzumab plus pertuzumab versus trastuzumab alone.

### 2.3. Neoadjuvant Treatment

Neoadjuvant treatment reflected routine clinical practice and the standards prevailing during the study period. For analysis, each regimen was coded according to the treatment actually delivered rather than the originally intended regimen.

The dominant treatment patterns were anthracycline- or fluorouracil-containing chemotherapy followed by a taxane with trastuzumab plus pertuzumab (AC/FEC-THP) or with trastuzumab alone (AC/FEC-TH). Other regimens included TCH, TCHP, paclitaxel plus trastuzumab, and paclitaxel plus trastuzumab and pertuzumab; infrequently used regimens were grouped as ‘other’.

Within the revised pretreatment HER2-positive cohort, 92 patients received trastuzumab plus pertuzumab and 20 received trastuzumab alone. Two patients did not receive neoadjuvant HER2-targeted therapy; because of the very small size of this category, it was retained for descriptive cohort reporting but was not included in the principal HER2-treatment comparison or multivariable models.

### 2.4. Pathological Assessment and Definition of Pathological Complete Response

Post-treatment pathological response was determined retrospectively from the original definitive surgical pathology reports generated as part of routine clinical care. No study-specific pathology re-review was performed. These pathological assessments preceded retrospective calculation of HALP and CT-derived body-composition parameters; therefore, the reporting pathologists were unaware of these study-specific variables when pathological response was determined.

The primary endpoint was pCR, defined as the absence of residual invasive carcinoma in both the breast and regional lymph nodes (ypT0/is ypN0); residual ductal carcinoma in situ was permitted. All other postoperative pathological findings were classified as residual disease.

### 2.5. Laboratory Parameters and HALP Score

Pretreatment laboratory data were obtained from routine blood tests performed during the 2 weeks before neoadjuvant systemic therapy. Hemoglobin, serum albumin, absolute lymphocyte count, platelet count, and absolute neutrophil count were recorded. If several eligible tests were available, the result nearest to treatment initiation was selected.

Hemoglobin and albumin were recorded in g/dL, while lymphocyte, neutrophil, and platelet counts were expressed as ×10^9^/L. HALP was calculated as [hemoglobin × albumin × absolute lymphocyte count × 100]/platelet count. Multiplication by 100 was used because hemoglobin and albumin were entered in g/dL rather than g/L [16].

Potential non-malignancy-related factors influencing individual HALP components, including infection or inflammation, anemia, corticosteroid exposure, recent transfusion, renal or hepatic dysfunction, nutritional deficiency, and concomitant medications, were not systematically captured in the retrospective dataset and therefore were not used as exclusion criteria or adjustment variables.

### 2.6. CT-Based Body Composition Analysis

Pretreatment CT imaging obtained before neoadjuvant therapy was reviewed for body-composition assessment. Measurements were performed on either contrast-enhanced diagnostic CT or the non-contrast CT component of PET/CT, according to image availability and adequacy. In the revised body-composition cohort, 61 patients were assessed using PET/CT-derived non-contrast CT and 27 using contrast-enhanced diagnostic CT. A single axial image at L3 was selected where both transverse processes were clearly visible.

All measurements were performed manually on the Sectra PACS workstation (Sectra AB, Linköping, Sweden). On the selected L3 section, the bilateral psoas major, erector spinae, quadratus lumborum, transversus abdominis, rectus abdominis, and internal and external oblique muscles were delineated separately.

Skeletal muscle was segmented using an attenuation range of −29 to +150 HU. Pixels from −190 to −30 HU, corresponding mainly to adipose tissue, and values > 150 HU, representing predominantly bone or calcification, were excluded from the muscle region.

Areas of the individual muscles were measured and then summed. For anatomical description, rectus abdominis, internal/external oblique, and transversus abdominis were grouped as the anterior compartment, while erector spinae, quadratus lumborum, and psoas major formed the posterior compartment. Their combined area represented total skeletal muscle area (SMA), reported in cm^2^.

SMI was calculated by dividing total L3 SMA by height squared (cm^2^/m^2^). The continuous SMI value was used in Model 1. Literature-based cutoffs of 38.5 and 41 cm^2^/m^2^ were additionally explored categorically.

Mean SMD was calculated across the complete segmented skeletal-muscle region. Myosteatosis was defined using the BMI-specific attenuation thresholds reported by Martin et al.: <41 HU in patients with BMI < 25 kg/m^2^ and <33 HU in those with BMI ≥ 25 kg/m^2^ [22]. An imaging-defined low-SMI obesity phenotype was defined operationally as the coexistence of low SMI (<41 cm^2^/m^2^) and obesity (BMI ≥ 30 kg/m^2^). Because muscle-strength and physical-performance measurements were unavailable in this retrospective cohort, this variable was not considered equivalent to the full contemporary clinical definition of sarcopenic obesity [15].

Patients without adequate L3 imaging contributed to analyses not requiring body-composition variables but were omitted from CT-based analyses.

### 2.7. Interobserver Reproducibility

Interobserver reproducibility was evaluated in 40 randomly selected patients. Two radiologists with 10 and 20 years of experience independently repeated the measurements on Sectra PACS while blinded to one another’s measurements.

Reliability was examined for total SMA, SMI, and mean muscle attenuation. ICCs with 95% confidence intervals were estimated using a two-way random-effects, absolute-agreement approach. Following Koo and Li [23], ICC values <0.50, 0.50–0.75, 0.75–0.90, and >0.90 were interpreted as poor, moderate, good, and excellent reliability, respectively.

### 2.8. Statistical Analysis

Analyses were conducted with IBM SPSS Statistics for Windows, version 23.0 (IBM Corp., Armonk, NY, USA), together with R (R Foundation for Statistical Computing, Vienna, Austria). Continuous variables are reported as mean ± standard deviation and median (minimum–maximum), and categorical variables as counts and percentages. Distributional assumptions were examined using Kolmogorov-Smirnov and Shapiro-Wilk tests. Between-group comparisons used the independent-samples *t* test for normally distributed data and the Mann-Whitney U test otherwise. Categorical associations were tested with Pearson chi-square; Yates-corrected chi-square, Fisher exact, or Monte Carlo Fisher exact tests were used when cell counts did not satisfy standard chi-square assumptions.

Interobserver measurement reliability was quantified with ICCs and 95% confidence intervals using a two-way random-effects model with absolute agreement, consistent with the framework proposed by Koo and Li [23].

Because several categorical predictors contained sparse cells and could produce separation with conventional logistic regression, factors associated with pCR were evaluated using Firth penalized logistic regression. Residual disease was coded as 0 and pCR as 1. Candidate predictors were first examined in separate univariable Firth models.

Two parsimonious multivariable models were subsequently constructed. Model 1 included continuous SMI, HALP score, hormone receptor status, clinical T category, clinical N category, HER2-treatment group, age, and pretreatment Ki-67. Model 2 replaced SMI with myosteatosis while retaining the same clinical covariates. To reduce parameter burden, clinical T category was dichotomized as T0–T2 versus T3–T4 and clinical nodal status as N0 versus N+. The imaging-defined low-SMI obesity phenotype was not included in the principal multivariable models because only four patients met this definition. Missing covariate values were not imputed. The principal source of missingness for the body-composition models was the absence of evaluable pretreatment L3 imaging, meaning that the primary imaging-derived exposure could not be directly reconstructed for those patients. Multiple imputation would therefore have required a strong and unverifiable missing-at-random assumption for the principal exposure and was not used. Multivariable analyses consequently used a complete-case approach and included only patients with available data for all model covariates. Both final models included 80 patients.

Model-level significance was evaluated using likelihood-ratio statistics, and Hosmer-Lemeshow testing was used to assess evidence of gross lack of fit. Because the multivariable models were fitted to examine adjusted associations rather than to derive a clinical prediction tool, discrimination and calibration measures were treated as secondary exploratory model diagnostics. These diagnostics included ROC AUC, precision-recall AUPRC, calibration intercept, calibration slope, Brier score, and apparent calibration plots; AUCs were compared using the DeLong method.

To quantify optimism in these secondary diagnostics, 1000 bootstrap resamples were used to obtain optimism-corrected AUC, AUPRC, calibration intercept, calibration slope, and Brier score. These bootstrap analyses were not intended to establish or validate a clinically deployable prediction model.

To explore potential confounding by treatment era, a sensitivity analysis added pertuzumab era (before versus March 2019 or later) as an additional covariate to both final Firth models. Because only one patient from the pre-March 2019 period remained in the complete-case modeling sample, treatment-era estimates were interpreted cautiously.

Because this was a retrospective fixed cohort, no a priori sample-size calculation was performed. A sensitivity power analysis was therefore conducted using G*Power v3.1.9.7 for logistic regression with the Demidenko variance-corrected large-sample *z* test. With a two-sided α of 0.05, 95% power, *n* = 80, baseline pCR probability of 0.55, R^2^ = 0, and a standardized continuous predictor distribution [X~N(0,1)], the detectable effect size corresponded to an OR of 0.394 in the decreasing direction and 2.537 in the increasing direction. Accordingly, smaller or moderate associations could not be reliably excluded. Because no clinically meaningful equivalence margin had been defined a priori, no TOST equivalence analysis was performed.

All tests were two-sided, with statistical significance defined as *p* < 0.05.

Generative AI assistance was used solely during manuscript preparation for language editing, readability, and refinement of academic presentation; no AI tool was used for data collection or statistical analysis.

### 2.9. Ethical Considerations

The retrospective clinical cohort included patients treated between March 2018 and December 2025. The December 2025 cutoff was selected to allow sufficient time for completion of neoadjuvant treatment, definitive surgery, and availability of postoperative pathology. The ethics application was submitted to the Non-Interventional Clinical Research Ethics Committee of Recep Tayyip Erdoğan University on 2 July 2026, and approval was granted on 27 August 2026 (Decision No. 2026/352). Study-specific retrospective data extraction was initiated only after ethical approval and was completed within approximately one week. CT-based skeletal-muscle measurements were subsequently completed over approximately two days. Statistical analyses were performed on 5 September 2026, and manuscript preparation was completed by 9 September 2026. Thus, no study-specific data extraction or statistical analysis was performed before ethical approval.

Only retrospectively collected institutional data were used. Identifying information was removed before analysis, and patient confidentiality was preserved throughout the study.

## 3. Results

### 3.1. Patient and Tumor Characteristics

The revised primary cohort comprised 114 patients with HER2-positive disease documented on pretreatment biopsy. Mean age at diagnosis was 54.60 ± 11.62 years (median, 55.5 years), and 67 patients (58.8%) were younger than 60 years. Forty-four patients (38.6%) were premenopausal and 70 (61.4%) postmenopausal. Mean BMI was 30.61 ± 6.81 kg/m^2^ (median, 29.13 kg/m^2^); 63 patients (55.3%) had BMI < 30 kg/m^2^ and 51 (44.7%) had BMI ≥ 30 kg/m^2^.

Invasive ductal carcinoma accounted for 96.5% of tumors. The mean largest pretreatment tumor diameter was 28.28 ± 15.69 mm (median, 25.0 mm). Clinically, 85 patients (74.6%) had T0–T2 disease and 29 (25.4%) had T3–T4 disease; 18 patients (15.8%) were node-negative and 96 (84.2%) node-positive. Histological grade was unavailable in 55 patients (48.2%). Hormone receptor-positive disease was present in 75 patients (65.8%), while 39 (34.2%) were hormone receptor-negative. Pretreatment Ki-67 data were available for 104 patients.

AC/FEC-THP was the most frequently administered neoadjuvant regimen (73.7%), followed by AC/FEC-TH (14.0%). With respect to HER2-directed therapy, 92 patients (80.7%) received trastuzumab plus pertuzumab, 20 (17.5%) received trastuzumab alone, and 2 (1.8%) received no neoadjuvant HER2-targeted therapy. Overall, 59 patients (51.8%) achieved pCR, whereas 55 (48.2%) had residual disease.

Body-composition measurements were evaluable in 88 patients. Mean skeletal muscle area was 114.93 ± 21.84 cm^2^, mean muscle attenuation was 22.13 ± 15.32 HU, and mean SMI was 45.07 ± 8.86 cm^2^/m^2^. Using the 41 cm^2^/m^2^ threshold, 31 patients (35.2%) had low SMI and 57 (64.8%) had high SMI. The imaging-defined low-SMI obesity phenotype was present in 4 patients (4.5%), whereas myosteatosis was identified in 72 (81.8%). Mean HALP score was 43.62 ± 17.81, with a median of 41.10. Baseline characteristics are summarized in Table 1.

### 3.2. Interobserver Reproducibility

Interobserver agreement between the two radiologists was excellent for all CT-derived skeletal-muscle measurements. The ICC was 0.970 (95% CI, 0.940–0.986) for total skeletal muscle area, 0.953 (95% CI, 0.858–0.981) for mean muscle attenuation, and 0.960 (95% CI, 0.920–0.980) for SMI; all *p* values were <0.001. Detailed reproducibility results are provided in Supplementary Table S1.

### 3.3. Factors Associated with Pathological Complete Response

Pathological response differed significantly according to hormone receptor status. pCR was achieved in 29 of 39 hormone receptor-negative tumors (74.4%) compared with 30 of 75 hormone receptor-positive tumors (40.0%; *p* < 0.001). No statistically significant association with pCR was detected for age group, menopausal status, BMI category, clinical T category, clinical N category, overall clinical stage, or Ki-67 category. Similarly, the simplified T0–T2 versus T3–T4 comparison (*p* = 0.387) and N0 versus N+ comparison (*p* = 1.000) were not significant.

Among patients included in the principal HER2-treatment comparison, pCR occurred in 52 of 92 patients (56.5%) who received trastuzumab plus pertuzumab and in 7 of 20 patients (35.0%) who received trastuzumab alone. This unadjusted difference did not reach statistical significance (*p* = 0.081). Treatment regimen showed a borderline overall association with pCR (*p* = 0.067).

Among the 88 patients with evaluable body-composition data, pCR occurred in 9 of 20 patients (45.0%) with SMI < 38.5 cm^2^/m^2^ and in 39 of 68 patients (57.4%) with SMI ≥ 38.5 cm^2^/m^2^ (*p* = 0.472). Using the 41 cm^2^/m^2^ threshold, pCR was observed in 13 of 31 patients (41.9%) with low SMI and in 35 of 57 patients (61.4%) with high SMI (*p* = 0.080). Myosteatosis was not significantly associated with pCR (*p* = 1.000). The imaging-defined low-SMI obesity phenotype was present in only four patients, precluding reliable inference regarding its association with pCR.

Imaging modality was not associated with pCR (*p* = 0.646). Likewise, pCR rates did not differ between patients treated before and after March 2019 (50.0% vs. 51.9%; *p* = 1.000). Categorical associations are summarized in Table 2.

When analyzed as continuous variables, age, pretreatment Ki-67, mean muscle attenuation, SMI, HALP, and BMI did not differ significantly between patients with residual disease and those who achieved pCR. Median SMI was 42.70 cm^2^/m^2^ in the residual-disease group and 44.70 cm^2^/m^2^ in the pCR group (*p* = 0.177). Median HALP was 41.12 and 40.44, respectively (*p* = 0.387).

On standalone ROC analysis, none of the host-related continuous variables showed strong discrimination for pCR. The AUC was 0.502 (95% CI, 0.379–0.624) for mean muscle attenuation, 0.584 (95% CI, 0.463–0.705) for SMI, and 0.545 (95% CI, 0.424–0.667) for HALP. Continuous-variable comparisons and standalone ROC analyses are summarized in Table 3.

### 3.4. Firth Penalized Logistic Regression Analyses

In univariable Firth analyses, hormone receptor positivity was associated with substantially lower odds of pCR compared with hormone receptor-negative disease (OR 0.239, 95% CI 0.099–0.540; *p* < 0.001). Trastuzumab alone, compared with trastuzumab plus pertuzumab, was associated with numerically lower odds of pCR, although this did not reach statistical significance in the full evaluable treatment cohort (OR 0.429, 95% CI 0.153–1.121; *p* = 0.085). Continuous SMI, categorical SMI thresholds of 38.5 and 41 cm^2^/m^2^, HALP, the imaging-defined low-SMI obesity phenotype, myosteatosis, simplified clinical T and N categories, age, and Ki-67 were not statistically significant in univariable models.

In Model 1, hormone receptor positivity remained associated with lower odds of pCR (OR 0.079, 95% CI 0.013–0.315; *p* < 0.001). Trastuzumab alone was also associated with lower pCR odds relative to trastuzumab plus pertuzumab (OR 0.044, 95% CI 0.003–0.266; *p* < 0.001). Continuous SMI was not statistically significantly associated with pCR (OR 1.077, 95% CI 0.997–1.176; *p* = 0.059), and HALP, clinical T and N categories, age, and Ki-67 were also not statistically significant.

In Model 2, myosteatosis replaced SMI while the same clinical covariates were retained. Hormone receptor positivity again remained associated with lower pCR odds (OR 0.082, 95% CI 0.015–0.317; *p* < 0.001), as did trastuzumab alone relative to trastuzumab plus pertuzumab (OR 0.037, 95% CI 0.003–0.234; *p* < 0.001). Myosteatosis was not independently associated with pCR (OR 0.452, 95% CI 0.105–1.766; *p* = 0.256). Age (OR 1.047, 95% CI 0.998–1.104; *p* = 0.061) and nodal positivity (OR 0.235, 95% CI 0.036–1.140; *p* = 0.073) were not statistically significant.

Both multivariable models were statistically significant overall (Model 1: LR χ^2^(8) = 31.714, *p* < 0.001; Model 2: LR χ^2^(8) = 29.575, *p* < 0.001; both *n* = 80). Hosmer-Lemeshow testing showed no evidence of gross lack of fit (Model 1: *p* = 0.912; Model 2: *p* = 0.489). Univariable and multivariable Firth results are shown in Table 4 and Table 5.

Both models were based on 80 complete cases. Model 1 included continuous SMI; Model 2 replaced SMI with myosteatosis. Clinical T and N categories were dichotomized as T0–T2 versus T3–T4 and N0 versus N+, respectively. The imaging-defined low-SMI obesity phenotype was not included because only four patients met the operational definition. The HER2-treatment coefficient is retained as an adjustment term for model transparency but should not be interpreted as a comparative treatment-effect estimate because the complete-case sample contained only 12 trastuzumab-alone patients, with one pCR event. Hosmer-Lemeshow testing showed no evidence of gross lack of fit.

### 3.5. Secondary Exploratory Model Diagnostics

As secondary exploratory diagnostics, the apparent AUC was 0.852 (95% CI, 0.771–0.932) for Model 1 and 0.835 (95% CI, 0.749–0.922) for Model 2. The difference between the two AUCs was not statistically significant by DeLong testing (difference 0.016, 95% CI −0.035 to 0.068; *p* = 0.530).

Apparent AUPRC was 0.886 for both models. After 1000 bootstrap resamples, optimism-corrected AUPRC values were 0.839 for Model 1 and 0.837 for Model 2.

Bootstrap-based optimism assessment reduced the AUC from 0.852 to 0.793 for Model 1 and from 0.835 to 0.770 for Model 2. Optimism-corrected calibration intercepts were −0.003 and 0.024, calibration slopes were 0.785 and 0.756, and Brier scores were 0.195 and 0.204 for Models 1 and 2, respectively. These diagnostics indicate residual optimism and overfitting and are presented only to characterize the fitted association models.

Apparent calibration plots and precision-recall curves are shown in Figure 2 and Figure 3, respectively; corresponding apparent and optimism-corrected exploratory diagnostics are summarized in Table 6.

Diagnostics were calculated in the complete-case analysis sample (*n* = 80). Bootstrap-based optimism correction used 1000 resamples. These measures are presented as exploratory diagnostics of the fitted association models and not as validation of a clinical prediction tool. DeLong comparison: ΔAUC = 0.016 (95% CI, −0.035 to 0.068); *p* = 0.530.

### 3.6. Sensitivity Analyses

To evaluate potential confounding by treatment era, pertuzumab era (before versus March 2019 or later) was added as an additional covariate to both final Firth models. Treatment era itself was not significantly associated with pCR in either model (Model 1: OR 0.274, 95% CI 0.005–59.035; *p* = 0.552; Model 2: OR 0.144, 95% CI 0.003–30.389; *p* = 0.384).

Adjustment for treatment era did not materially alter the estimated associations for hormone receptor status, SMI, HALP, myosteatosis, or HER2-treatment group. The OR for trastuzumab alone relative to trastuzumab plus pertuzumab changed from 0.044 to 0.046 in Model 1 and from 0.037 to 0.038 in Model 2. However, only one patient from the pre-March 2019 period remained in the final complete-case sample of 80 patients. Accordingly, these sensitivity analyses had limited ability to disentangle treatment-era effects from HER2-treatment selection. Detailed results are provided in Supplementary Table S2.

### 3.7. Impact of CT Acquisition Protocol on Muscle Attenuation and Myosteatosis

Because body-composition measurements were derived from both the non-contrast CT components of PET/CT and contrast-enhanced diagnostic CT, we examined whether acquisition type influenced attenuation-based muscle metrics. Among the 88 patients with evaluable body-composition data, myosteatosis was present in 58 of 61 patients (95.1%) assessed using PET/CT-derived CT compared with 14 of 27 patients (51.9%) assessed using diagnostic CT (*p* < 0.001).

Mean skeletal muscle attenuation was significantly lower in the PET/CT group than in the diagnostic CT group (18.50 ± 11.91 HU vs. 30.33 ± 18.89 HU; *p* = 0.005). In contrast, mean SMI was nearly identical between acquisition groups (45.15 ± 8.91 vs. 44.90 ± 8.92 cm^2^/m^2^; *p* = 0.904). These findings demonstrated a significant association between imaging acquisition type and both muscle attenuation and myosteatosis classification, whereas SMI did not differ by acquisition type. These results are summarized in Table 7.

## 4. Discussion

In this retrospective cohort of 114 patients with pretreatment HER2-positive breast cancer receiving neoadjuvant systemic therapy, we did not detect statistically significant associations between pCR and pretreatment SMI, myosteatosis, the imaging-defined low-SMI obesity phenotype, or HALP score. Hormone receptor status showed a strong association with pathological response. HER2-treatment group was also associated with pCR in the multivariable models; however, this coefficient should not be interpreted as a comparative treatment-effect estimate because of the small trastuzumab-alone subgroup, the composition of the complete-case sample, and the strong relationship between treatment choice and treatment era. Overall, the host-related body-composition and inflammatory-nutritional measures evaluated here were not independently associated with pCR.

For skeletal muscle quantity, neither categorical nor continuous SMI analyses reached statistical significance. Using the 41 cm^2^/m^2^ cutoff, pCR was observed in 61.4% of patients with higher SMI and 41.9% of those with lower SMI (*p* = 0.080). In univariable Firth regression, SMI ≥ 41 cm^2^/m^2^ yielded an OR of 2.162 (95% CI 0.905–5.289; *p* = 0.083), and continuous SMI in multivariable Model 1 yielded an OR of 1.077 per cm^2^/m^2^ (95% CI 0.997–1.176; *p* = 0.059). Because the confidence intervals included the null value and the prespecified significance threshold was not met, these findings do not establish an independent association between SMI and pCR.

Published evidence regarding skeletal muscle mass and neoadjuvant response in breast cancer remains heterogeneous. Günaltılı et al. reported lower pCR rates among patients with low SMI [9], whereas Butler et al., using a 38.5 cm^2^/m^2^ threshold in a broader neoadjuvant breast cancer population, did not demonstrate a statistically significant association with pCR [10]. Karaca et al. similarly reported no independent association between sarcopenia and pathological response in locally advanced breast cancer [24], while Işıklar et al. observed no statistically significant difference in pCR [25]. Differences in breast cancer subtype, treatment regimen, body-composition definitions, imaging methodology, and sample size may contribute to these inconsistencies. In the present study, body-composition measurements were available for 88 patients, and the sensitivity power analysis indicated that the final multivariable sample had adequate power only for relatively large effects. This limits precision for smaller effects but does not alter the interpretation that an independent association between SMI and pCR was not demonstrated in the present cohort.

Myosteatosis was not significantly associated with pCR in either univariable or multivariable analyses. However, interpretation of this finding is substantially limited by imaging heterogeneity. Myosteatosis was present in 95.1% of patients assessed using PET/CT-derived CT compared with 51.9% of those assessed using diagnostic CT, and mean muscle attenuation was significantly lower in the PET/CT group (18.50 vs. 30.33 HU; *p* = 0.005). By contrast, mean SMI was almost identical between acquisition groups (45.15 vs. 44.90 cm^2^/m^2^; *p* = 0.904). These findings suggest that attenuation-based muscle measures were considerably more sensitive to CT acquisition characteristics than muscle-area-based SMI in this cohort. In addition, the Martin et al. attenuation thresholds were not developed or validated specifically in a Turkish HER2-positive breast cancer population with a relatively high mean BMI; therefore, population-specific misclassification cannot be excluded. Accordingly, the high overall prevalence of myosteatosis (81.8%) should not be interpreted solely as a biological characteristic of the study population.

Only four patients met the operational definition of the imaging-defined low-SMI obesity phenotype. Given this very small subgroup, the absence of a statistically significant association with pCR cannot be meaningfully interpreted. Contemporary definitions of sarcopenic obesity incorporate not only reduced muscle mass and excess adiposity but also measures of muscle strength or physical performance [15]. Because such functional assessments were unavailable in this retrospective cohort, we deliberately use the term imaging-defined low-SMI obesity phenotype rather than sarcopenic obesity. This variable was therefore retained for descriptive purposes but excluded from the principal multivariable models.

HALP likewise did not show a statistically significant association with pCR. Median HALP values were similar in patients with residual disease and those achieving pCR (41.12 vs. 40.44; *p* = 0.387), and standalone discrimination was limited (AUC 0.545, 95% CI 0.424–0.667). HALP was also non-significant in both multivariable models. These findings should not be interpreted as evidence that systemic inflammatory-nutritional status has no relationship with treatment response. Rather, they indicate that conventional pretreatment HALP did not provide strong standalone discrimination for pCR in this cohort. HALP has shown prognostic and response-related associations in breast cancer [17,18,19,20], but statistical association and discriminatory performance are distinct concepts. Accordingly, significant associations reported in larger or differently selected cohorts do not necessarily imply strong classification performance, and the present AUC of 0.545 should be interpreted in that context rather than as directly contradictory to the prior literature. Moreover, individual HALP components may be influenced by factors unrelated to tumor biology, including infection or inflammation, anemia, corticosteroid exposure, recent transfusion, renal or hepatic dysfunction, nutritional status, and concomitant medications. These potential influences were not systematically captured in the present retrospective dataset and residual confounding cannot be excluded.

Hormone receptor status showed the most consistent association with pathological response. pCR was achieved in 74.4% of hormone receptor-negative tumors compared with 40.0% of hormone receptor-positive tumors (*p* < 0.001). This inverse association for hormone receptor positivity remained significant in both multivariable Firth models (Model 1: OR 0.079, 95% CI 0.013–0.315; Model 2: OR 0.082, 95% CI 0.015–0.317; both *p* < 0.001). These findings are consistent with prior neoadjuvant HER2-positive studies showing higher pCR rates in hormone receptor-negative disease and reinforce the importance of intrinsic tumor biology in determining response [1,4,6].

HER2-directed treatment group was retained in the multivariable models as a clinically relevant adjustment variable, but its coefficient should not be interpreted as an estimate of comparative treatment effectiveness. In the full evaluable treatment cohort, pCR occurred in 56.5% of patients receiving trastuzumab plus pertuzumab and 35.0% of those receiving trastuzumab alone, with the unadjusted comparison not reaching statistical significance (*p* = 0.081). In the final complete-case sample, however, only 12 patients received trastuzumab alone and only one achieved pCR, compared with 43 of 68 patients treated with dual HER2 blockade. This near-degenerate event distribution produced an unstable and markedly amplified adjusted treatment coefficient. Accordingly, the treatment-group ORs are presented for model completeness only and should not be used to infer relative treatment efficacy.

Treatment era was strongly related to HER2-treatment selection, with trastuzumab-alone therapy used more frequently in the earlier period and dual blockade predominating after March 2019. Although addition of treatment era to the multivariable models did not materially change the HER2-treatment OR estimates, this should not be interpreted as excluding era-related confounding. In the final complete-case sample, only one patient remained from the pre-March 2019 period, making reliable separation of treatment-era effects from treatment selection impossible. Accordingly, the large adjusted ORs for HER2-treatment group should be interpreted as observational associations rather than causal treatment effects. Residual confounding by treatment era, patient selection, and other unmeasured clinical factors cannot be excluded.

Secondary exploratory diagnostics showed apparent AUCs of 0.852 and 0.835, which decreased to 0.793 and 0.770 after bootstrap-based optimism correction. Calibration slopes were also below 1 after correction, indicating residual overfitting. These quantities are reported to characterize model behavior in the present dataset; they do not establish clinical prediction performance or utility, and the fitted models should be regarded as exploratory multivariable association models rather than prediction tools.

This study has several methodological strengths. It evaluated multiple host-related domains—skeletal muscle quantity, muscle attenuation, the imaging-defined low-SMI obesity phenotype, and HALP—within the same HER2-positive neoadjuvant cohort. Body-composition measurements were performed manually at L3 across the full skeletal-muscle compartment, with excellent interobserver reproducibility. Firth penalized logistic regression was used to reduce bias related to sparse data and separation, the final models were simplified to reduce parameter burden, and bootstrap-based optimism assessment was used as a secondary diagnostic of model behavior. The potential influence of treatment era and CT acquisition protocol was also specifically examined.

Several limitations should be acknowledged. The retrospective single-center design introduces the possibility of selection bias, residual confounding, and limited generalizability. Although the primary cohort included 114 patients, body-composition data were available for 88 and the final multivariable models included 80 complete cases. This reduction in sample size limited precision and may also have introduced complete-case selection bias. We did not use multiple imputation because the dominant missing component was the imaging-derived body-composition exposure itself in patients without evaluable pretreatment L3 imaging; imputing this exposure would have depended on an unverifiable missing-at-random assumption and would not have resolved the sparse treatment-event distribution. In particular, only 12 trastuzumab-alone patients remained in the complete-case sample, with a single pCR event, and only one patient from the pre-March 2019 period remained. These imbalances prevent reliable separation of treatment-group effects from treatment-era and selection effects and substantially limit interpretation of the corresponding OR estimates.

Imaging heterogeneity represents an additional limitation. Body-composition measurements were derived from both PET/CT-derived CT and contrast-enhanced diagnostic CT. Muscle attenuation and myosteatosis prevalence differed substantially by acquisition type, whereas SMI did not, indicating that attenuation-based measures were particularly sensitive to protocol differences. In addition, only four patients met the imaging-defined low-SMI obesity phenotype, precluding meaningful inference for this subgroup. Potential non-cancer-related determinants of HALP components were not systematically recorded and therefore could not be adjusted for. Finally, the secondary bootstrap diagnostics demonstrated residual optimism and overfitting; these findings further support avoiding interpretation of the fitted models as clinically validated prediction tools.

Because this was a retrospective fixed cohort, no a priori sample-size calculation was performed. The sensitivity analysis indicated that the final multivariable sample could detect only relatively large effects with 95% power; smaller or moderate associations could therefore not be reliably excluded.

Taken together, the present findings do not support an independent association between pretreatment SMI, myosteatosis, the imaging-defined low-SMI obesity phenotype, or HALP and pCR in this cohort. Protocol sensitivity of attenuation-based measures and residual confounding in treatment-related analyses further limit interpretation. Larger prospective studies using standardized imaging protocols and more complete clinical characterization are needed to determine whether host-related body-composition or inflammatory-nutritional markers provide reproducible information beyond established tumor-biological factors.

## 5. Conclusions

In this retrospective cohort of patients with pretreatment HER2-positive early or locally advanced breast cancer, no statistically significant associations were detected between pCR and pretreatment SMI, myosteatosis, the imaging-defined low-SMI obesity phenotype, or HALP score. Hormone receptor status remained associated with pathological response. The observed association between HER2-treatment group and pCR should not be interpreted as a comparative treatment-effect estimate because of sparse complete-case subgroup data and potential residual confounding by treatment era. Attenuation-based muscle measures were sensitive to CT acquisition type, limiting interpretation of myosteatosis. Larger prospective studies using standardized imaging protocols and adequately powered cohorts are needed to determine whether body-composition and inflammatory-nutritional markers provide reproducible information beyond established clinicopathological factors.

## Figures and Tables

**Figure 1:**
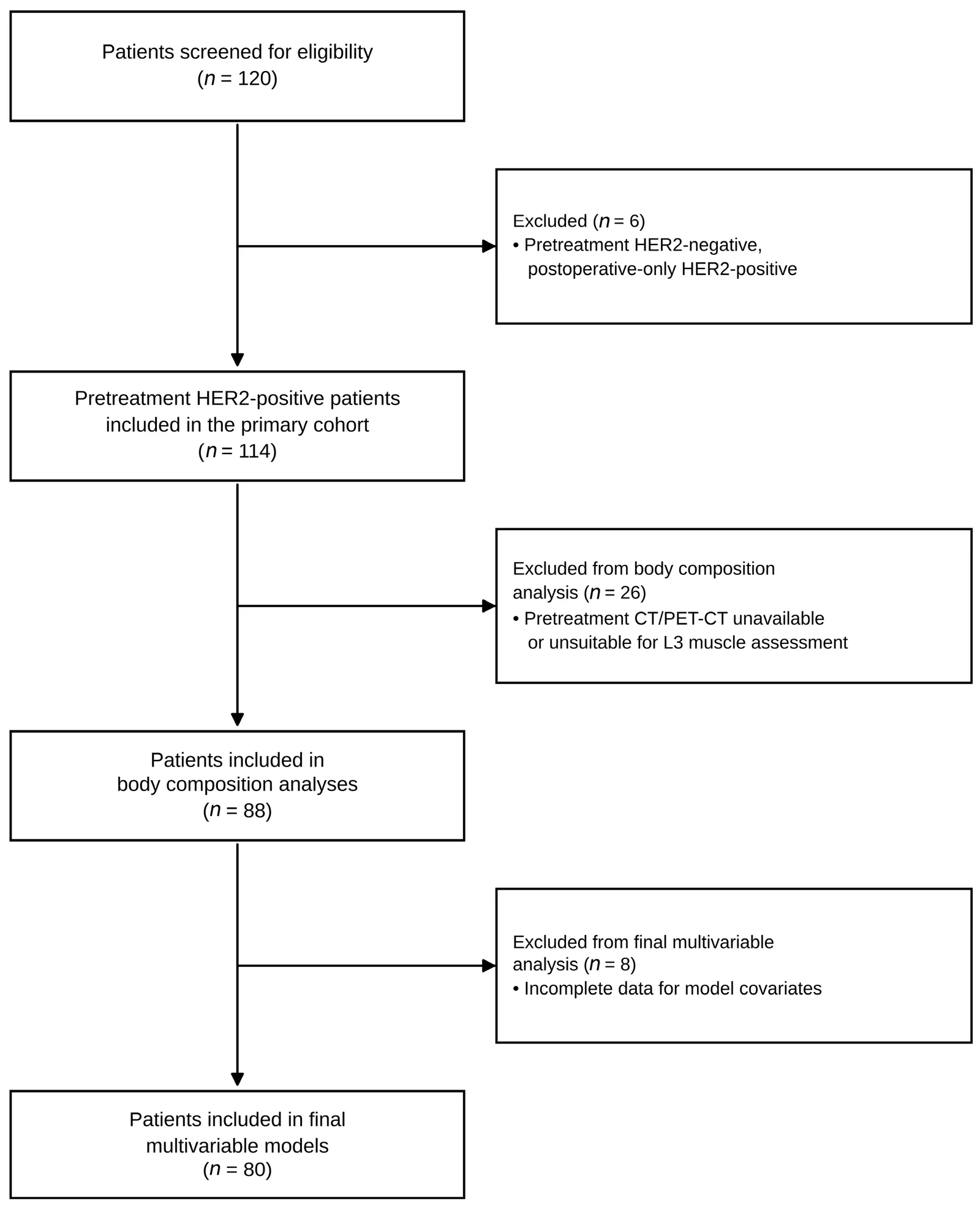
Flow diagram of patient selection and analysis cohorts. Of 120 initially identified patients, six patients who were HER2-negative on pretreatment biopsy and HER2-positive only in residual tumor at surgery were excluded from the primary cohort. The resulting primary cohort included 114 patients with pretreatment HER2-positive disease; 88 had evaluable pretreatment CT or PET/CT images for body-composition assessment, and 80 had complete data for the final multivariable models. CT, computed tomography; HER2, human epidermal growth factor receptor 2; L3, third lumbar vertebra; PET/CT, positron emission tomography/computed tomography.

**Figure 2:**
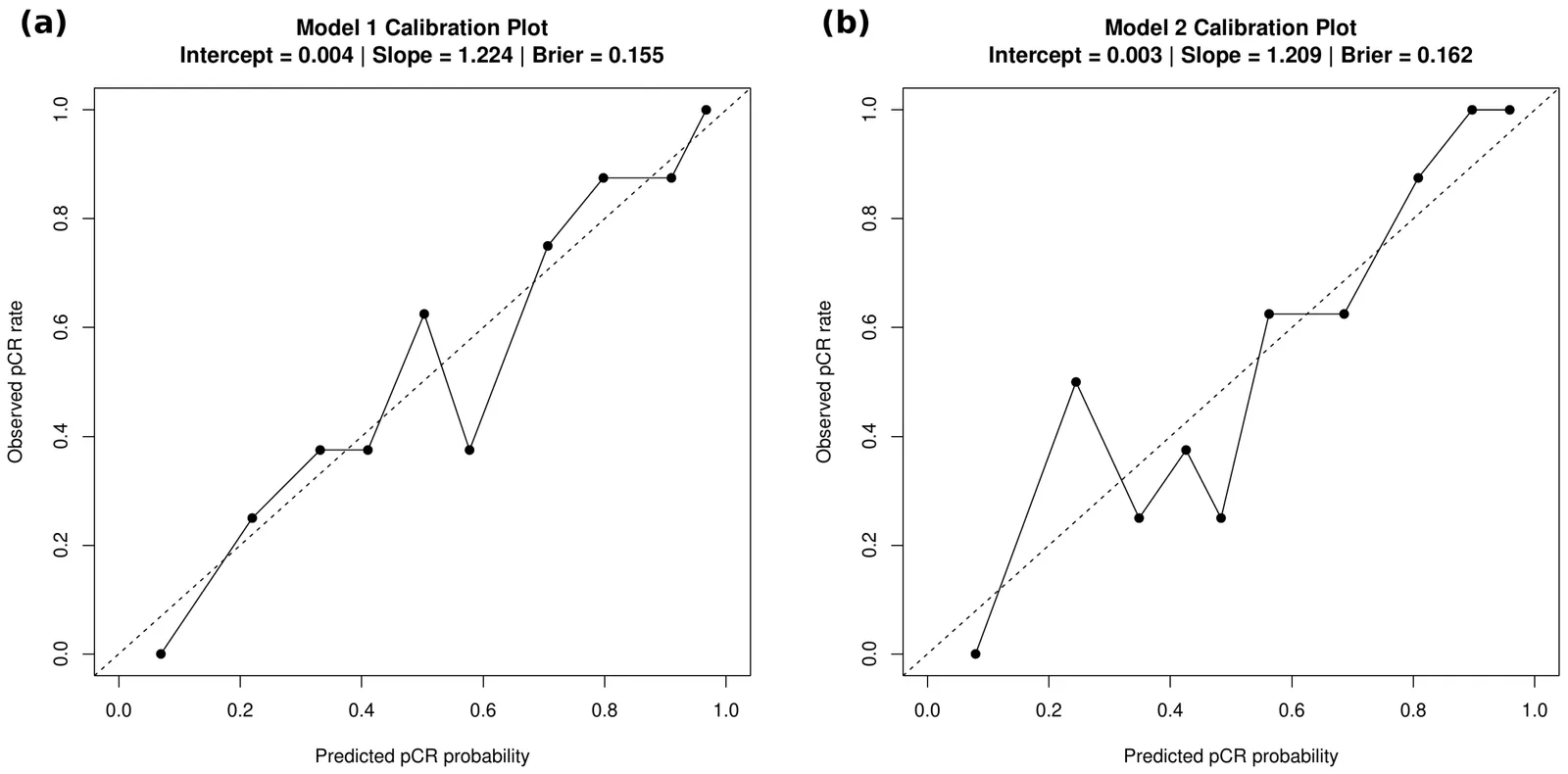
Apparent calibration plots of the final Firth penalized logistic regression models, presented as secondary exploratory diagnostics. (**a**) Model 1, including continuous skeletal muscle index; (**b**) Model 2, including myosteatosis. The dashed diagonal line represents perfect calibration, whereas the solid line represents the observed relationship between predicted and observed probabilities of pathological complete response in the complete-case analysis sample. Values shown within each panel represent apparent calibration metrics. Optimism-corrected diagnostics derived from 1000 bootstrap resamples are reported in Table 6.

**Figure 3:**
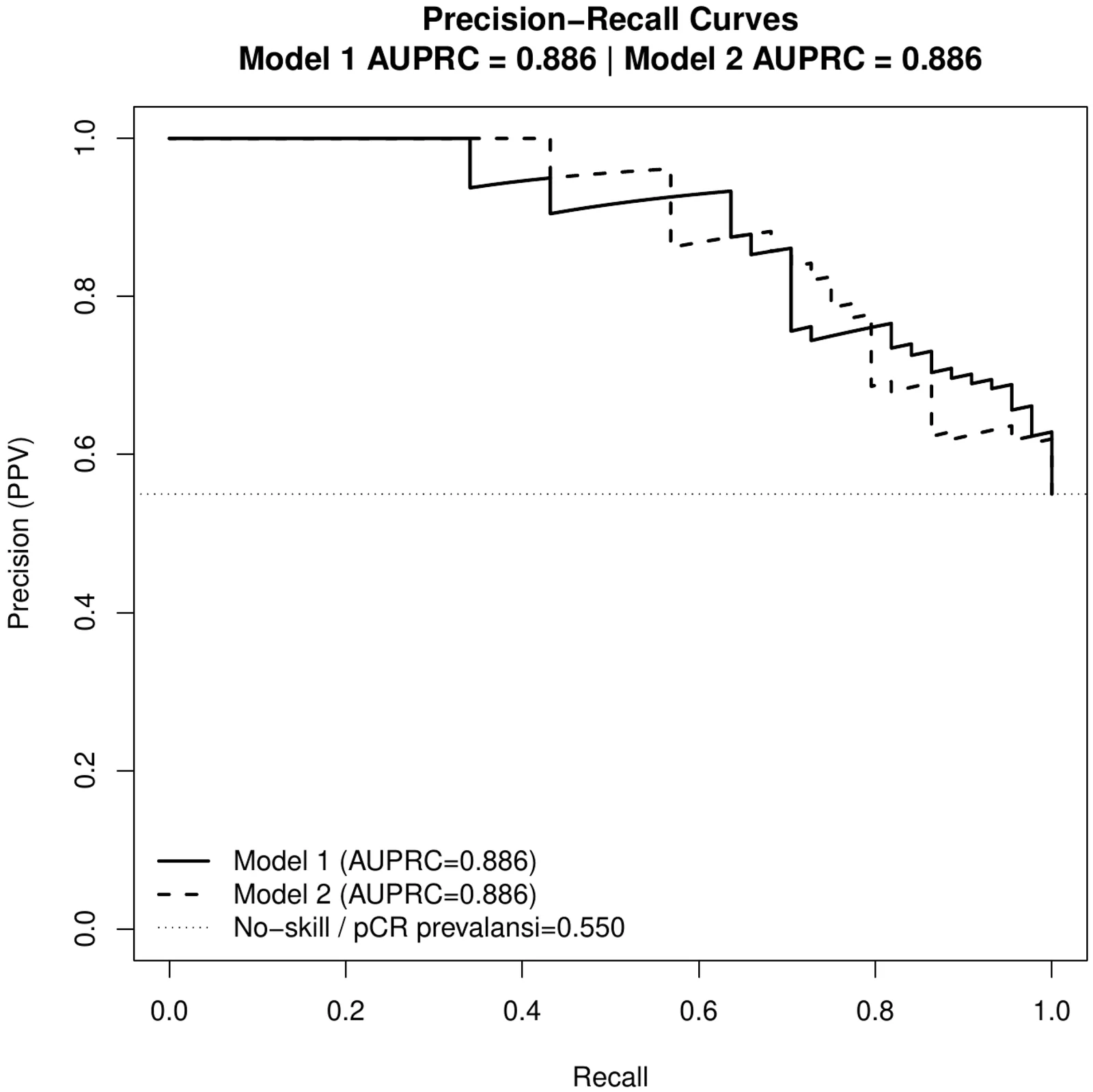
Apparent precision-recall curves of the final Firth penalized logistic regression models, presented as secondary exploratory diagnostics. Model 1 and Model 2 both yielded an apparent area under the precision-recall curve (AUPRC) of 0.886 in the complete-case analysis sample. The horizontal dashed line represents the pCR prevalence in the analysis sample (0.550) and serves as the no-skill reference. Optimism-corrected AUPRC values derived from 1000 bootstrap resamples are reported in Table 6.

**Table 1:** Clinical, pathological, treatment, and body-composition characteristics of the study cohort.

Characteristic	Available *n*	Value
Age at diagnosis, years	114	54.60 ± 11.62; 55.50 (28.00–79.00)
<60 years	114	67 (58.8)
≥60 years	114	47 (41.2)
Premenopausal	114	44 (38.6)
Postmenopausal	114	70 (61.4)
BMI, kg/m^2^	114	30.61 ± 6.81; 29.13 (16.95–49.98)
BMI < 30 kg/m^2^	114	63 (55.3)
BMI ≥ 30 kg/m^2^	114	51 (44.7)
PET/CT	114	77 (67.5)
Diagnostic CT	114	37 (32.5)
Invasive ductal carcinoma	114	110 (96.5)
Invasive lobular carcinoma	114	3 (2.6)
Mixed histology	114	1 (0.9)
Largest pretreatment tumor diameter, mm	114	28.28 ± 15.69; 25.00 (0.00–90.00)
Clinical T0	114	1 (0.9)
Clinical T1	114	34 (29.8)
Clinical T2	114	50 (43.9)
Clinical T3	114	4 (3.5)
Clinical T4	114	25 (21.9)
Clinical T0–T2	114	85 (74.6)
Clinical T3–T4	114	29 (25.4)
Clinical N0	114	18 (15.8)
Clinical N1	114	77 (67.5)
Clinical N2	114	12 (10.5)
Clinical N3	114	7 (6.1)
Clinical N+	114	96 (84.2)
Clinical stage IA	114	5 (4.4)
Clinical stage IIA	114	38 (33.3)
Clinical stage IIB	114	31 (27.2)
Clinical stage IIIA	114	10 (8.8)
Clinical stage IIIB	114	23 (20.2)
Clinical stage IIIC	114	7 (6.1)
Grade 1	114	1 (0.9)
Grade 2	114	37 (32.5)
Grade 3	114	21 (18.4)
Grade unknown	114	55 (48.2)
Hormone receptor-negative	114	39 (34.2)
Hormone receptor-positive	114	75 (65.8)
HER2 IHC 2+	114	20 (17.5)
HER2 IHC 3+	114	94 (82.5)
Ki-67, %	104	31.75 ± 15.10; 30.00 (4.00–80.00)
Ki-67 <30%	104	46 (44.2)
Ki-67 ≥30%	104	58 (55.8)
HALP score	112	43.62 ± 17.81; 41.10 (5.72–104.96)
AC/FEC-TH	114	16 (14.0)
AC/FEC-THP	114	84 (73.7)
TCH	114	1 (0.9)
TCHP	114	5 (4.4)
Paclitaxel + trastuzumab	114	3 (2.6)
Paclitaxel + trastuzumab + pertuzumab	114	3 (2.6)
Other regimen	114	2 (1.8)
Trastuzumab + pertuzumab	114	92 (80.7)
Trastuzumab alone	114	20 (17.5)
No neoadjuvant HER2-targeted therapy	114	2 (1.8)
Before March 2019	114	8 (7.0)
March 2019 or later	114	106 (93.0)
Residual disease	114	55 (48.2)
pCR	114	59 (51.8)
SMA, cm^2^	88	114.93 ± 21.84; 112.18 (70.67–174.78)
SMD, HU	88	22.13 ± 15.32; 22.08 (−12.04–64.06)
SMI, cm^2^/m^2^	88	45.07 ± 8.86; 43.89 (29.04–72.75)
SMI < 41 cm^2^/m^2^	88	31 (35.2)
SMI ≥ 41 cm^2^/m^2^	88	57 (64.8)
Myosteatosis	88	72 (81.8)
Imaging-defined low-SMI obesity phenotype	88	4 (4.5)

Data are presented as *n* (%) or mean ± standard deviation; median (minimum–maximum), as appropriate. Percentages were calculated using the number of patients with available data for the respective variable. Body-composition measurements were available for 88 patients, Ki-67 for 104 patients, and HALP for 112 patients. Abbreviations: BMI, body mass index; CT, computed tomography; HALP, hemoglobin-albumin-lymphocyte-platelet; HER2, human epidermal growth factor receptor 2; IHC, immunohistochemistry; pCR, pathological complete response; PET/CT, positron emission tomography/computed tomography; SMA, skeletal muscle area; SMD, skeletal muscle radiodensity; SMI, skeletal muscle index.

**Table 2:** Associations between categorical clinicopathological, treatment, and body-composition variables and pathological complete response.

Variable	Residual Disease, *n* (%)	pCR, *n* (%)	*p* Value
Age < 60 years	31 (46.3)	36 (53.7)	0.614
Age ≥ 60 years	24 (51.1)	23 (48.9)	
Premenopausal	20 (45.5)	24 (54.5)	0.636
Postmenopausal	35 (50.0)	35 (50.0)	
BMI < 30 kg/m^2^	32 (50.8)	31 (49.2)	0.545
BMI ≥ 30 kg/m^2^	23 (45.1)	28 (54.9)	
Clinical T0	0 (0.0)	1 (100.0)	0.909
Clinical T1	16 (47.1)	18 (52.9)	
Clinical T2	23 (46.0)	27 (54.0)	
Clinical T3	2 (50.0)	2 (50.0)	
Clinical T4	14 (56.0)	11 (44.0)	
Clinical T0–T2	39 (45.9)	46 (54.1)	0.387
Clinical T3–T4	16 (55.2)	13 (44.8)	
Clinical N0	9 (50.0)	9 (50.0)	0.803
Clinical N1	35 (45.5)	42 (54.5)	
Clinical N2	7 (58.3)	5 (41.7)	
Clinical N3	4 (57.1)	3 (42.9)	
Clinical N0 (binary)	9 (50.0)	9 (50.0)	1.000
Clinical N+	46 (47.9)	50 (52.1)	
Clinical stage IA	4 (80.0)	1 (20.0)	0.520
Clinical stage IIA	15 (39.5)	23 (60.5)	
Clinical stage IIB	15 (48.4)	16 (51.6)	
Clinical stage IIIA	4 (40.0)	6 (60.0)	
Clinical stage IIIB	13 (56.5)	10 (43.5)	
Clinical stage IIIC	4 (57.1)	3 (42.9)	
Hormone receptor-negative	10 (25.6)	29 (74.4)	<0.001
Hormone receptor-positive	45 (60.0)	30 (40.0)	
Ki-67 < 30%	22 (47.8)	24 (52.2)	0.826
Ki-67 ≥ 30%	29 (50.0)	29 (50.0)	
AC/FEC-TH	10 (62.5)	6 (37.5)	0.067
AC/FEC-THP	36 (42.9)	48 (57.1)	
TCH	1 (100.0)	0 (0.0)	
TCHP	4 (80.0)	1 (20.0)	
Paclitaxel + trastuzumab	2 (66.7)	1 (33.3)	
Paclitaxel + trastuzumab + pertuzumab	0 (0.0)	3 (100.0)	
Other regimen	2 (100.0)	0 (0.0)	
Trastuzumab + pertuzumab	40 (43.5)	52 (56.5)	0.081
Trastuzumab alone	13 (65.0)	7 (35.0)	
Imaging-defined low-SMI obesity phenotype absent	38 (45.2)	46 (54.8)	1.000
Present	2 (50.0)	2 (50.0)	
Myosteatosis absent	7 (43.8)	9 (56.2)	1.000
Myosteatosis present	33 (45.8)	39 (54.2)	
SMI < 38.5 cm^2^/m^2^	11 (55.0)	9 (45.0)	0.472
SMI ≥ 38.5 cm^2^/m^2^	29 (42.6)	39 (57.4)	
SMI < 41 cm^2^/m^2^	18 (58.1)	13 (41.9)	0.080
SMI ≥ 41 cm^2^/m^2^	22 (38.6)	35 (61.4)	
PET/CT	36 (46.8)	41 (53.2)	0.646
Diagnostic CT	19 (51.4)	18 (48.6)	
Before March 2019	4 (50.0)	4 (50.0)	1.000
March 2019 or later	51 (48.1)	55 (51.9)	

The principal HER2-treatment comparison included trastuzumab plus pertuzumab versus trastuzumab alone; two patients who received no neoadjuvant HER2-targeted therapy were excluded from this inferential comparison. For multicategory variables, the *p* value shown on the first row represents the overall group comparison. Pearson chi-square, Yates-corrected chi-square, Fisher exact, or Monte Carlo Fisher exact tests were used as appropriate. Percentages are row percentages.

**Table 3:** Continuous variables according to pathological response and standalone discrimination for pCR.

Panel A. Variable	Residual Disease		pCR		*p* Value
Age at diagnosis, years	59.00 (28.00–79.00), *n* = 55		54.00 (34.00–77.00), *n* = 59		0.798
Ki-67, %	30.00 (4.00–80.00), *n* = 51		30.00 (10.00–75.00), *n* = 53		0.824
SMD, HU	22.51 ± 16.34, *n* = 40		21.81 ± 14.58, *n* = 48		0.832
SMI, cm^2^/m^2^	42.70 (29.04–72.75), *n* = 40		44.70 (30.32–67.86), *n* = 48		0.177
HALP score	41.12 (11.46–104.96), *n* = 53		40.44 (5.72–101.66), *n* = 59		0.387
BMI, kg/m^2^	28.65 (20.44–49.98), *n* = 55		29.62 (16.95–46.67), *n* = 59		0.669
**Panel B. Variable**		**AUC (95% CI)**		***p* Value**	
SMD, HU		0.502 (0.379–0.624)		0.980	
SMI, cm^2^/m^2^		0.584 (0.463–0.705)		0.177	
HALP score		0.545 (0.424–0.667)		0.466	

Data are presented as mean ± standard deviation or median (minimum–maximum), as appropriate. Between-group comparisons used the independent-samples *t* test or Mann-Whitney U test. AUC values were obtained from standalone ROC analyses for pCR.

**Table 4:** Univariable Firth penalized logistic regression analyses for pathological complete response.

Predictor	*n*	OR (95% CI)	*p* Value
SMI, per 1 cm^2^/m^2^ increase	88	1.028 (0.981–1.082)	0.251
SMI ≥ 38.5 cm^2^/m^2^	88	1.621 (0.607–4.417)	0.334
SMI ≥ 41 cm^2^/m^2^	88	2.162 (0.905–5.289)	0.083
HALP score, continuous	112	1.009 (0.988–1.031)	0.402
Imaging-defined low-SMI obesity phenotype, present vs. absent	88	0.828 (0.123–5.592)	0.838
Myosteatosis, present vs. absent	88	0.931 (0.313–2.691)	0.895
Hormone receptor-positive vs. negative	114	0.239 (0.099–0.540)	<0.001
Clinical T3–T4 vs. T0–T2	114	0.695 (0.298–1.599)	0.392
Clinical N+ vs. N0	114	1.086 (0.401–2.939)	0.869
Trastuzumab alone vs. trastuzumab + pertuzumab	112	0.429 (0.153–1.121)	0.085
Age at diagnosis, per year	114	0.998 (0.967–1.030)	0.884
Ki-67 ≥ 30% vs. <30%	104	0.918 (0.425–1.979)	0.828

ORs were estimated using separate univariable Firth penalized logistic regression models with pCR coded as the event. The HER2-treatment analysis excluded the two patients who received no neoadjuvant HER2-targeted therapy.

**Table 5:** Final multivariable Firth penalized logistic regression models for pathological complete response.

Predictor	Model 1 OR (95% CI)	*p* Value	Model 2 OR (95% CI)	*p* Value
SMI, per 1 cm^2^/m^2^ increase	1.077 (0.997–1.176)	0.059	—	—
Myosteatosis, present vs. absent	—	—	0.452 (0.105–1.766)	0.256
HALP score, continuous	0.988 (0.951–1.023)	0.503	0.989 (0.954–1.023)	0.536
Hormone receptor-positive vs. negative	0.079 (0.013–0.315)	<0.001	0.082 (0.015–0.317)	<0.001
Clinical T3–T4 vs. T0–T2	0.597 (0.163–2.101)	0.422	0.877 (0.253–3.075)	0.835
Clinical N+ vs. N0	0.326 (0.052–1.515)	0.157	0.235 (0.036–1.140)	0.073
Trastuzumab alone vs. trastuzumab + pertuzumab	0.044 (0.003–0.266)	<0.001	0.037 (0.003–0.234)	<0.001
Age at diagnosis, per year	1.023 (0.973–1.079)	0.379	1.047 (0.998–1.104)	0.061
Ki-67 ≥ 30% vs. <30%	0.532 (0.153–1.654)	0.281	0.701 (0.225–2.061)	0.522

**Table 6:** Secondary exploratory discrimination and calibration diagnostics of the final multivariable models.

Performance Metric	Model 1	Model 2
Apparent AUC	0.852	0.835
95% CI for apparent AUC	0.771–0.932	0.749–0.922
AUC optimism	0.059	0.066
Optimism-corrected AUC	0.793	0.770
Apparent AUPRC	0.886	0.886
AUPRC optimism	0.046	0.050
Optimism-corrected AUPRC	0.839	0.837
Optimism-corrected calibration intercept	−0.003	0.024
Optimism-corrected calibration slope	0.785	0.756
Optimism-corrected Brier score	0.195	0.204

**Table 7:** CT-derived body-composition measures according to imaging acquisition type.

Variable	PET/CT-Derived CT (*n* = 61)	Diagnostic CT (*n* = 27)	*p* Value
SMD, HU	18.50 ± 11.91	30.33 ± 18.89	0.005
SMI, cm^2^/m^2^	45.15 ± 8.91	44.90 ± 8.92	0.904
Myosteatosis, *n* (%)	58 (95.1)	14 (51.9)	<0.001

Data are presented as mean ± standard deviation or *n* (%), as appropriate. PET/CT-derived CT refers to the non-contrast CT component of PET/CT, whereas diagnostic CT refers to contrast-enhanced diagnostic CT. These results are summarized in Table 7.

## Data Availability

The data are available from the corresponding author upon reasonable request, subject to institutional and ethical restrictions.

## Supplementary Materials

The following supporting information can be downloaded at: https://www.mdpi.com/article/10.3390/jcm15207927/s1, Table S1: Interobserver reproducibility of CT-derived skeletal-muscle measurements; Table S2: Sensitivity analyses including treatment era as an additional covariate in the final Firth penalized logistic regression models; Table S3: Distribution of pathological response according to HER2-targeted treatment group in the evaluable treatment cohort and final complete-case modeling sample; Supplementary files S1: STROBE Cohort Checklist–Completed Mapping.
