# Supplementary material for: Pretreatment Body Composition Parameters and HALP Score in Relation to Pathological Complete Response in HER2-Positive Breast Cancer Receiving Neoadjuvant Systemic Therapy
Source: J Clin Med. 2026 Oct 14;15(20):7927. doi: 10.3390/jcm15207927 (PMC13706024; doi:10.3390/jcm15207927)
Supplement: Supplementary file 1 [file jcm-15-07927-s001.zip › Supplementary File S1 STROBE Cohort Checklist – Completed Mapping.pdf]

STROBE Cohort Checklist – Completed Mapping

Pretreatment Body Composition Parameters and HALP Score in Relation to Pathological Complete Response in HER2-Positive Breast Cancer Receiving Neoadjuvant Systemic Therapy

Manuscript ID: jcm-4586543

This completed mapping follows the item numbering and domains of the STROBE cohort-study checklist. Reporting requirements are paraphrased; page numbers refer to the final second-revision manuscript.

Item	Domain	Reporting requirement (paraphrased)	Location in manuscript
1a	Title/abstract	Identify observational design in title or abstract.	Abstract, p. 1
1b	Title/abstract	Provide balanced summary of methods and findings.	Abstract, p. 1
2	Background/rationale	Explain scientific background and rationale.	Introduction, p. 2
3	Objectives	State objective and associations of interest.	Introduction, p. 2
4	Study design	Present key design elements early.	Methods 2.1, pp. 2–3
5	Setting	Describe center, period, and relevant dates.	Methods 2.1, 2.9, pp. 2–3, 7–8
6a	Participants	Describe eligibility, exclusions, and selection.	Methods 2.1; Fig. 1, pp. 2–4
6b	Participants	Report matching criteria for matched studies.	N/A; unmatched cohort
7	Variables	Define outcome, exposures, covariates, criteria.	Methods 2.2–2.6, pp. 4–6
8	Data sources/measurement	Describe sources and measurement methods.	Methods 2.2–2.7, pp. 4–6
9	Bias	Describe efforts to address potential bias.	Methods 2.1, 2.8; Discussion, pp. 2–3, 7, 16–19
10	Study size	Explain how study size was determined.	Methods 2.8, p. 7
11	Quantitative variables	Explain handling/categorization.	Methods 2.2, 2.6, 2.8, pp. 4–7
12a	Statistical methods	Describe methods and confounder adjustment.	Methods 2.8, pp. 6–7
12b	Subgroups/interactions	Describe subgroup/interaction methods if used.	Sensitivity analyses: Methods 2.8; Results 3.6–3.7, pp. 7, 15–16
12c	Missing data	Explain handling of missing data.	Methods 2.8; limitations, pp. 7, 18
12d	Loss to follow-up	Explain handling if applicable.	N/A; endpoint was postoperative pCR
12e	Sensitivity analyses	Describe sensitivity analyses.	Methods 2.8; Results 3.6–3.7, pp. 7, 15–16
13a	Participants	Report numbers at each study stage.	Methods 2.1; Fig. 1; Results 3.1, pp. 3–4, 8
13b	Participants	Give reasons for exclusions/non-participation.	Methods 2.1; Fig. 1, pp. 3–4
13c	Participants	Consider use of a flow diagram.	Figure 1, p. 4
14a	Descriptive data	Report characteristics and confounders.	Table 1; Results 3.1, pp. 8–9
14b	Descriptive data	Report numbers with missing data.	Tables 1–7; Suppl. Table S3, pp. 8–16
14c	Descriptive data	Summarize follow-up time.	N/A; primary endpoint was pCR after surgery
15	Outcome data	Report outcome-event numbers/summary measures.	Results 3.1–3.3; Tables 1–3, pp. 8–11
16a	Main results	Report unadjusted/adjusted estimates with precision and adjustment variables.	Results 3.3–3.4; Tables 3–5, pp. 10–13
16b	Main results	Report cut points for categorized continuous variables.	Methods 2.2, 2.6, 2.8; Tables 1–5, pp. 4–13
16c	Main results	Translate relative estimates to absolute risk when relevant.	N/A; no time-based relative-risk estimate
17	Other analyses	Report other analyses, sensitivity, diagnostics.	Results 3.2, 3.5–3.7, pp. 9, 13–16
18	Key results	Summarize key results relative to objectives.	Discussion, pp. 16–18
19	Limitations	Discuss major bias/imprecision.	Discussion, pp. 17–19
20	Interpretation	Provide cautious overall interpretation.	Discussion, pp. 16–19
21	Generalisability	Discuss external validity/generalizability.	Discussion, pp. 18–19
22	Funding	State funding source and funder role.	Funding statement, p. 19
