# Supplementary material for: Pretreatment Body Composition Parameters and HALP Score in Relation to Pathological Complete Response in HER2-Positive Breast Cancer Receiving Neoadjuvant Systemic Therapy
Source: J Clin Med. 2026 Oct 14;15(20):7927. doi: 10.3390/jcm15207927 (PMC13706024; doi:10.3390/jcm15207927)
Supplement: Supplementary file 1 [file jcm-15-07927-s001.zip › Supplementary_Material_Tables.pdf]

Supplementary Material

Pretreatment Body Composition Parameters and HALP Score in Relation to Pathological Complete Response in HER2-Positive Breast Cancer Receiving Neoadjuvant Systemic Therapy

Table S1. Interobserver reproducibility of CT-derived skeletal-muscle measurements.

Measurement	ICC	95% CI	p value
SMA, mm²	0.970	0.940–0.986	<0.001
SMA, cm²	0.970	0.940–0.985	<0.001
SMD / mean muscle attenuation, HU	0.953	0.858–0.981	<0.001
SMI, cm²/m²	0.960	0.920–0.980	<0.001

ICCs were estimated using a two-way random-effects model with absolute agreement in a randomly selected subset of 40 patients. Abbreviations: CI, confidence interval; CT, computed tomography; HU, Hounsfield unit; ICC, intraclass correlation coefficient; SMA, skeletal muscle area; SMD, skeletal muscle radiodensity; SMI, skeletal muscle index.

Table S2. Sensitivity analyses including treatment era as an additional covariate in the final Firth penalized logistic regression models.

Predictor	Model 1 OR (95% CI)	p value	Model 2 OR (95% CI)	p value
SMI, per 1 cm²/m² increase	1.077 (0.997–1.176)	0.059	—	—
Myosteatosis, present vs. absent	—	—	0.454 (0.105–1.766)	0.258
HALP score, continuous	0.988 (0.951–1.023)	0.498	0.989 (0.954–1.023)	0.534
Hormone receptor-positive vs. negative	0.080 (0.013–0.315)	<0.001	0.082 (0.015–0.317)	<0.001
Clinical T3–T4 vs. T0–T2	0.602 (0.163–2.101)	0.429	0.880 (0.253–3.075)	0.840
Clinical N+ vs. N0	0.331 (0.052–1.515)	0.163	0.238 (0.036–1.140)	0.075
Trastuzumab alone vs. trastuzumab + pertuzumab	0.046 (0.004–0.296)	<0.001	0.038 (0.003–0.250)	<0.001
Treatment era: March 2019 or later vs. before March 2019	0.274 (0.005–59.035)	0.552	0.144 (0.003–30.389)	0.384
Age at diagnosis, per year	1.023 (0.973–1.079)	0.376	1.047 (0.998–1.104)	0.061
Ki-67 ≥30% vs. <30%	0.537 (0.153–1.654)	0.288	0.704 (0.225–2.061)	0.526

Both sensitivity models included 80 complete cases and nine predictors. Model 1: likelihood-ratio $\chi^2(9)=31.114$, $p<0.001$; Hosmer-Lemeshow p=0.904. Model 2: likelihood-ratio $\chi^2(9)=28.944$, $p=0.001$; Hosmer-Lemeshow p=0.488. Only one patient from the pre-March 2019 period remained in the complete-case modeling sample; therefore, treatment-era estimates are highly imprecise and should not be interpreted as evidence that era-related confounding is absent. Abbreviations: CI, confidence interval; HALP, hemoglobin-albumin-lymphocyte-platelet; OR, odds ratio; SMI, skeletal muscle index.

Table S3. Distribution of pathological response according to HER2-targeted treatment group in the evaluable treatment cohort and final complete-case modeling sample.

Analysis sample	HER2-targeted treatment	Residual disease, n	pCR, n	Total, n
Evaluable treatment cohort (n=112)	Trastuzumab + pertuzumab	40	52	92
	Trastuzumab alone	13	7	20
Final complete-case modeling sample (n=80)	Trastuzumab + pertuzumab	25	43	68
	Trastuzumab alone	11	1	12

The two patients who received no neoadjuvant HER2-targeted therapy were not included in the principal treatment comparison. The marked change in treatment-group composition in the complete-case sample, particularly the single pCR event among 12 trastuzumab-alone patients, should be considered when interpreting the adjusted treatment effect estimates. Abbreviations: HER2, human epidermal growth factor receptor 2; pCR, pathological complete response.
